# Proposed Standards for Variable Harmonization Documentation and Referencing: A Case Study Using QuickCharmStats 1.1

**DOI:** 10.1371/journal.pone.0147795

**Published:** 2016-02-09

**Authors:** Kristi Winters, Sebastian Netscher

**Affiliations:** International Data Infrastructures, GESIS Leibniz Institute for the Social Sciences, Köln, Nordrhein-Westfalen, Germany; Iowa State University, UNITED STATES

## Abstract

Comparative statistical analyses often require data harmonization, yet the social sciences do not have clear operationalization frameworks that guide and homogenize variable coding decisions across disciplines. When faced with a need to harmonize variables researchers often look for guidance from various international studies that employ output harmonization, such as the Comparative Survey of Election Studies, which offer recoding structures for the same variable (e.g. marital status). More problematically there are no agreed documentation standards or journal requirements for reporting variable harmonization to facilitate a transparent replication process. We propose a conceptual and data-driven digital solution that creates harmonization documentation standards for publication and scholarly citation: QuickCharmStats 1.1. It is free and open-source software that allows for the organizing, documenting and publishing of data harmonization projects. QuickCharmStats starts at the conceptual level and its workflow ends with a variable recording syntax. It is therefore flexible enough to reflect a variety of theoretical justifications for variable harmonization. Using the socio-demographic variable ‘marital status’, we demonstrate how the CharmStats workflow collates metadata while being guided by the scientific standards of transparency and replication. It encourages researchers to publish their harmonization work by providing researchers who complete the peer review process a permanent identifier. Those who contribute original data harmonization work to their discipline can now be credited through citations. Finally, we propose peer-review standards for harmonization documentation, describe a route to online publishing, and provide a referencing format to cite harmonization projects. Although CharmStats products are designed for social scientists our adherence to the scientific method ensures our products can be used by researchers across the sciences.

## Introduction

Harmonizing data for statistical analysis is a routine aspect of scientific research; the BioSHaRE (Biobank Standardisation and Harmonisation for Research Excellence in the European Union) project is but one such example [1]. Large scale social surveys such as the European Values Survey publish extensive documentation that describes its variable harmonizations [2]. However, when faced with the need to do original ex-post harmonization researchers must spend time investigating the documentation of various international studies that employ output harmonization; in practice that means searching through perhaps hundreds of pages of text to find specific recoding information. The result is that multiple researchers spend time searching through extensive documentation looking up the same recoding notes. Researchers that cannot find a harmonization structure to replicate must do the best they can to harmonize the response options in the target and source variables. Journal articles rarely allow authors the word count to fully report their harmonization recoding in detail and the recording syntaxes themselves are rarely published as an appendix. It is not difficult to see the problems of trying to compare the findings of three different researchers who independently attempted to replicate a previous study’s harmonized variable structure; each could end up recreating the same work without knowing whether they have recoded the harmonization identically to the original study or to each other’s. This is a waste of resources, time and effort, and the absence of the original recoding syntax is a barrier to the scientific principles of transparency and replicability. We see the lack of common standards for documenting and reporting the method of variable harmonization as a main driver of these problems. To solve this problem we present our software solution to establish standards of best practices and peer review for variable harmonization documentation, publication and referencing. In this we aim to start the conversation about guidelines for the documentation of data harmonization, and discuss what information is both necessary and sufficient to replicate another’s work.

Another obstacle to precise replication in variable harmonization is that the social sciences do not share a single, overarching theoretical framework that guides and homogenizes variable harmonization coding decisions across disciplines. Further, such an overarching theoretical framework for the social sciences might not be desirable since each discipline brings its own theoretical application of a concept to a variable. For instance, while a political scientist might see little need to separate out couples that cohabitate from couples who are married when using marital status as a control variable, sociologists or economists might want to use the same measure but disambiguate cohabitation from marriage informed by social norms or tax implications in order to capture variation. A theoretically flexible variable harmonization tool is therefore desirable.

To address these gaps in social science practices and fill lacuna in digital software solutions we present the free and open-source desktop software QuickCharmStats 1.1 (QCS). GESIS—Leibniz Institute for the Social Sciences has developed QSC for researchers who harmonize variables for use in published statistical analyses. CharmStats–an abbreviation of ‘Coding and Harmonization of Statistics’–products allow for the importation and combining of multiple types of metadata along with any additional information used in harmonizing. QuickCharmStats is atheoretical in its structure and instead starts with concepts, moves to operationalization of the target and source variables, facilitates recoding within its workflow and in the end generates the data recoding syntax in the statistical languages of SPSS and Stata. Its workflow ensures transparency through the careful documentation of the harmonization process, including information on the variable name, label and its values, the question wording, and information about the source studies. Space is provided to note any literature or publications that guided the recoding and allows researchers to make notes on the logic behind coding decisions that might not be obvious. All the information from a project can be published as a report, and it is this report that is submitted for peer review and published with a DOI at GESIS.

Below we review the finding that increasing numbers of journals have established or are establishing data availability policies. These policies require that data be made publically available for any published research; however, providing the data does not provide researchers with the ability to precisely replicate variable harmonization recoding since the documentation is not part of the requirement. Our product fills the lacuna between data availability and access to precise information on variable harmonization. We present a QCS harmonization report as an example of our proposed peer-review standards and what information is to be submitted to GESIS for review and publication. We use the socio-demographic variable ‘marital status’ from the British Election Study (BES) and harmonize it to the Comparative Study of Electoral Systems (CSES). We chose this measure because harmonizing the response categories for marital status can be confirmed using face validity by specialists and non-specialists alike. For completeness we conclude by proposing a referencing guideline for published harmonizations.

QuickCharmStats arrives as conditions are right for a digital tool to enhance scientific rigor while reducing the time and effort involved in providing maximal transparency in variable recoding and harmonization. Driving the increased pressure to document variable harmonizations are: more and better quality data available to harmonize; increasingly sophisticated statistical methods used to analyze said data; and the specific and transparent information required to replicate variables, thus advancing scientific theories. Not only is replication key to evaluating the quality of a piece of research, but it allows new research paths to be developed and explored [3, 4]. After testing prior conclusions that data-based findings are becoming more frequent in the social sciences, Gherghina and Katsanidou noted that having access to the data itself is necessary [4–6]. Gherghina and Katsanidou investigated and assessed the data availability policies of several international, peer-reviewed political science journals and the content of those policies. Their analysis confirmed that journals in political science have increasingly adopted data availability policies thereby improving the overall replication standards discipline-wide. Given that they found higher numbers of journals adopting such policies as compared with previous studies, the authors predict the number of journals adopting data availability policies will continue to increase.

The scientific ideal of providing all data and information necessary to replicate findings accurately is confronted by the reality that, although programming advances have increased the scale and sophistication of statistical analyses, research articles must often omit many methodological details due to space or stylistic constraints [7]. Even the obvious solution of including the syntax with the article does not solve the problem. That is because:

1. there are no conventions that outline how the syntax code should be published (i.e. SPSS syntax or Stata do files);
2. syntax files alone may not contain the information necessary to replicate others’ work (i.e. if questions were answered based on prior routing); and
3. merely providing the syntax does not provide insight into why certain coding decisions were made and how those decisions influenced the variable recoding.

In spite of all this, precise replication of the original study’s variable transformations is necessary for an exact reproduction of the analysis. Such precise replication requires a high degree of transparency from the original researcher. On the subject of transparency, Moravcsik writes,

‘Transparency is the cornerstone of social science. Academic discourse rests on the obligation of scholars to reveal to their colleagues the data, theory, and methodology on which their conclusions rest. Unless other scholars can examine evidence, parse the analysis, and understand the processes by which evidence and theories were chosen, why should they trust—and thus expend the time and effort to scrutinize, critique, debate, or extend—existing research?’ [8]

Given the importance of transparency to precise replication and truly comparable statistical results, the lack of scientific standards for documenting and reporting variable harmonizations is a serious obstacle to advancements in our understanding. Our first CharmStats product deals with and overcomes the practical obstacles to transparent variable harmonization with a tool that provides theoretical flexibility in defining and operationalizing key concepts for use in statistical analysis.

## Discussion

### QuickCharmStats rationale and technical information

CharmStats is the name of a line of harmonization software products in development by Martin Friedrichs at GESIS—Leibniz Institute for the Social Sciences. QCS was the first product released and it is available for download on the CharmStats website. It is desktop software with a specific audience in mind: researchers working on their own computers, who use SPSS or Stata to conduct their statistical analyses, and who have fewer than one hundred variables to harmonize for any one research project. The primary goals are to 1) reduce the time it takes to assemble the metadata needed to accurately document the harmonization work; 2) improve data management by housing information used to make coding decisions in the same location as metadata on the variables, questions and studies and the SPSS and Stata recoding syntaxes, and 3) provide researchers with the template to generate a submission form to have their harmonization peer reviewed and published so they may cite it in the bibliography of their research articles.

Rather than impose a theoretical framework on all researchers a CharmStats project starts with the conceptual level, allowing the researchers to define their concepts guided by the most appropriate theory, or to document the creation of competing measures of a concept guided by alternative theories. For this reason QCS guides users through a logical workflow to import and work with all the metadata necessary to fully document variable harmonizations. Once the necessary metadata has been imported and organized users may click a button to generate harmonization syntax in both SPSS and Stata, creating reports that can be saved in a folder as either.html or.pdf, and a graph generator that displays (*inter alia*) source and target response mapping for visual inspection. Fields are provided to attach extended notes on the coding decision process, e.g. how a researcher dealt with missing cases in a particular nation or why she coded ‘Don’t know’ responses as valid, rather than missing, data.

**Fig 1:**
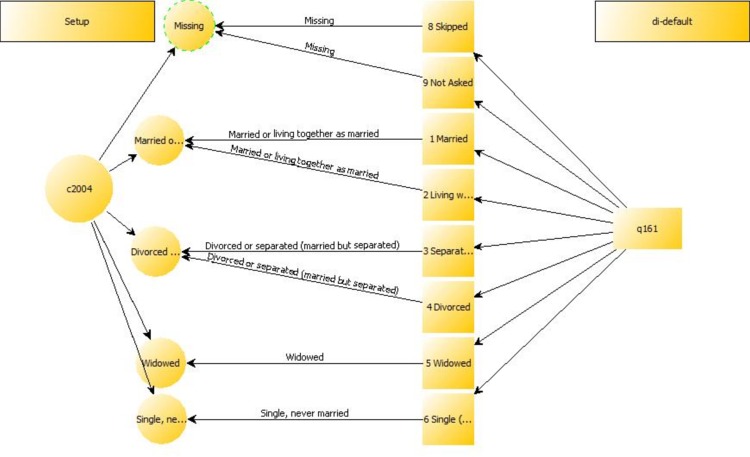
Marital Status harmonization mapping.

This article is also an attempt to set out what information is both necessary and sufficient to accurately replicate another’s harmonization work. Drawing on personal experience with ex-post harmonization on an international comparative survey we determined the following metadata were necessary to report in order for someone else to have sufficient information to recreate the harmonized variable: 1) the name of the project and its author(s) so the work is searchable and findable, 2) the syntax for the harmonization coding in both SPSS and Stata for precise replication, 3) information on the purpose of the harmonization, 4) the name of the concept to be operationalized and harmonized, as well as any literature or previous work that informs the recoding, 5) metadata on the target and source variables including name, label, definition, the response options values and their labels as well as the level of measurement and whether it is individual or aggregate level data, 6) metadata on the target and source question wording, including notes on the questions and relevant instructions to interviewers on how the question was to be administered, and 7) metadata on the name of the study and its permanent identifier or online source for the data (Table 1 and Fig 1). This information is provided so future researchers can consult the original study’s documentation for details that may be important (sampling frame, collection dates, mode of data collection) but are not necessary to replicate the harmonization recoding.

**Table 1:** Proposed format for Harmonization Project Documentation using CharmStats.

**Project name:**	Marital Status Comparative Study of Electoral Systems Module 3 to British Election Study 2010
**Project submitted date:**	10 December 2015
**Project published date:**	22 December 2015
**Author:**	Winters, Kristi
**Author:**	Netscher, Sebastian
**Harmonization syntax**	
**SPSS syntax:**	RECODE Q161 (8 = SYSMIS) (9 = SYSMIS) (1 = 1.00) (2 = 1.00) (3 = 3.00) (4 = 3.00) (5 = 2.00) (6 = 4.00) INTO c2004.VARIABLE LABELS c2004 ' new harmonized variable '. EXECUTE.
**Stata syntax:**	generate c2004 = q161 recode c2004 (8 =.) (9 =.) (1 = 1) (2 = 1) (3 = 3) (4 = 3) (5 = 2) (6 = 4) label variable c2004 "new harmonized variable"
**Project**	
**Project notes:**	This project harmonizes the CSES Module 3 (2006–2011) variable 'Marital status' with the British Election Study 2010 variable 'Marital status'.
**Project end use notes:**	**-**
**Concept**	
**Concept name:**	Marital status
**Definition:**	Marital status refers to whether the respondent is married or not. Expansion of marital status categories beyond ‘single’ and ‘married’ include ‘widow/er’ ‘divorced’ and ‘separated’. Cohabitation refers to two people, not married, who live together in an intimate relationship on a long-term basis.
**Concept comments:**	Both surveys harmonized here provide response options that conflate the concepts of marital status and cohabitation in this measurement. These categories do not capture same-sex civil partnerships or changes in same-sex marriage laws.
**Literature:**	**-**
**Target Variable**	
**Target name:**	c2004
**Target label:**	Marital or Civil Union Status
**Sampling level:**	Individual / Microdata
**Measurement level:**	Nominal
**Target question wording:**	Respondent’s marital or civil union status
**Response options and coding:**	
	7.00:Refused
	8.00:Don't know
	9.00:Missing
	1.00:Married or living together as married
	2.00:Widowed
	3.00:Divorced or separated (married but separated)
	4.00:Single, never married
	5.00:(See election notes)
**Source Variable**	
**Source name:**	q161
**Source label:**	Marital Status
**Sampling level:**	Individual / Microdata
**Measurement level:**	Nominal
**CS Pro instance name:**	di-default
**Source question wording:**	What is your marital status? Are you:
**Response options and coding:**	
	8:Skipped
	9:Not Asked
	1:Married
	2:Living with a partner
	3:Separated (after being married)
	4:Divorced
	5:Widowed
	6:Single (never married)
**Source definition:**	Question wording taken from http://bes.utdallas.edu/2009/bes-data/cses/MEMOCSES.pdf. Note that the BES CSES survey is a free-standing national post-election internet survey. It is not a subset of questions asked in any other BES survey.
**Source variable dataset:**	The British Election Study http://www.bes2009-10.org/ 2010 BES CSES DATA 12 May to 3 Jun-2010, Great Britain., GB YouGov Panel members Sample size is N = 927. Internet panel Weights: The standard YouGov weight is W8. Two other weights are also available.
**Date last accessed:**	July 29 2014.
**Source variable comments:**	‘The questionnaire includes the CSES Module 3 questions in the order suggested by the CSES planning committee. Also included are some standard BES questions, e.g., the BES party identification question, to facilitate comparisons of results produced by CSES and BES measures of key variables.’ http://bes.utdallas.edu/2009/bes-data/cses/MEMOCSES.pdf

QuickCharmStats is a Java® based desktop application. All CharmStats software products work by storing persistent information in a relational database. QCS works on a local database instance (localhost). The MySQL DBMS was chosen as the system to manage the database and its content. MySQL (Structured Query Language) is an open-source relational database management system (RDBMS) owned by Oracle Corporation. The software supports versions of Java 1.6 or higher and users can download MySQL software for free if they do not have it. The installation package for QCS is available from the GESIS website [9]. Users must provide a name and email address, then a link for the download is sent to their email accounts. The QCS software, its code, the user manual [10] and practice datasets come as part of the download. After copying the zip-file to the place of installation and unzipping it a user must connect QCS to the MySQL database. This is done by running a setup batch file in the windows command processor (cmd.exe) once. To start the application, double-click the CStatsApp jar-file symbol in the QuickCharmStats directory. The program is ready for use. QCS was developed for PC; however, Mac-tested versions are planned for the future.

QCS was designed for use by social researchers doing statistical analyses. Given their popularity, syntax for SPSS and Stata were selected as the first languages to be generated in the 1.0 and 1.1 versions. Both software packages were examined to evaluate their performance as a source of metadata. To date we have not found a way to retrieve variable metadata from Stata but we have using SPSS; therefore QCS 1.1 imports data using SPSS datasets (.sav files). It is important to note that a CharmStats user need not own the rights to use SPSS in order to use.sav datasets to import metadata into CharmStats. The.sav is read by CharmStats without using the SPSS software. Stata users can use Stat/Transfer to export their datasets as.sav files for importation into QuickCharmStats. Researchers without access to Stat/Transfer can email their.dta file to charmstats@gesis.org at least 2 weeks in advance of when they require the.sav file, free of charge. The number of syntax languages will be expanded in later versions (e.g. SAS). The Data Documentation Initiative (DDI) is an international effort to create a standard to describe statistical and social science data, and work is underway on the QCS 1.2 version that will allow metadata to be imported using forms of DDI (3.0 and 3.1). However, since more social researchers use SPSS or Stata data files to conduct their analyses we decided to realize the.sav import feature first, to make the most of users existing skill sets and to realize the DDI import functions in future versions of the software.

### Harmonization documentation types

In our view, the first step in establishing common harmonization documentation practices within and across scientific disciplines is to determine the necessary and sufficient information required to accurately replicate prior variable harmonization work. Unlike other publications a harmonization project is not a single piece of work. Researchers must combine information from one target variable (the harmonized variable structure) and at least one source variable (the original, unharmonized variable). This could mean a researcher needs to report metadata for one target variable in the target dataset, and at least one but potentially dozens of source variables from different source datasets. Therefore, CharmStats products collect and organize information on many levels of metadata to streamline the process and ensure replicability of the data harmonization. We review how the software organizes metadata and other information below.

### Types of information hand-entered by the user

- *Project information*: Harmonizations in CharmStats are organized into projects. A project contains various levels of metadata information and the response option mapping associated with harmonizing *N* source variables to a single target variable.
- *Concept*: At its most abstract, variable harmonization is an attempt to harmonize concepts. A project contains the work associated with harmonizing one concept using one target and at least one source variable. QSC 1.1 facilitates single dimension concept harmonization, such as socio-demographic variables. However, the CharmStats data model allows for the harmonization of composite variables such as scales. This capability will be realized in later versions of the software.

### Metadata collected or hand-entered during the data importation process

- *Target and source variables*: Target and source variable metadata contain similar information. The variable name, label, values and value labels, and measurement level are all imported from the.sav file.
- *Question information* Metadata on the question can be included as well. To facilitate replication and reuse researchers should include the question wording, interviewer instructions, skip pattern structures, or other information needed to replicate the work.
- *Study information*: QuickCharmStats allows users to attach the same study-level metadata to a series of variables while they are importing them. The name and version of the study, its persistent identifier (e.g. the DOI), the primary investigator(s), data collection methods, field period and collection mode are typed in once and then linked to each variable from that dataset.

#### Software outputs

QCS integrates different levels of metadata: on the study, the question and the variable itself. Over the course of its workflow users link this metadata as well as connect the source response options to the target harmonization structure using dropdown boxes. In short, QCS documents all necessary metadata to ensure transparency in the harmonization process and to enable replicability of the harmonized data. QCS collates this information to produce the following:

- *Syntax*: Recoding syntax for each target and source variable. Users can open a dialog that automatically generates syntax for SPSS and Stata.
- *Report*: The report dialog generates reports with all relevant metadata. QCS comes with templates for creating reports or users can create their own templates using the key words recognized by the software (listed in an appendix of the user manual). These reports can be saved as.html or converted into.pdf.
- *Graphs*: Similar to the automatic syntax feature, the graph feature generates a visual representation of the target and source response options, a map showing how the source and target variable responses were recoding, as well as other elements in the workflow. Graphs can be embedded into Reports.

To demonstrate QCS in use we present the digital harmonization project report (Table 1) and its graph (Fig 1). The report was generated from metadata used to harmonize ‘marital status’ as measured in the British Election Study (BES) 2010 with the Comparative Study of Electoral Systems (CSES) ‘marital status’ variable. First we summarize the two studies and explain how the data harmonization gap emerged. We then review the CSES harmonization procedures. Finally, we explain how QCS fills this gap by organizing the metadata and generating standardized harmonization syntaxes in SPSS and Stata.

### The CSES, the BES and the data gap

The Comparative Study of Electoral Systems (CSES) is a research project that collects and harmonizes post-election studies from around the world. Since 1996, the CSES has harmonized and published more than 180 post-election studies from 55 different countries covering various political topics [11]. It requires that samples must be random and cover the voting population in the participating countries. Data may be collected using: face-to-face interviews, telephone interviews or self-administrated mail-back questionnaires. This is done to ensure comparability as well as similar data quality across election studies. Uniformity in these standards can be traced back to the first CSES data collection in 1996; a time when online-sampling was uncommon, internet penetration was limited as well as highly skewed, and web-based survey were a novel method of data collection. These data collection requirements remain in effect until the third module which is why the CSES databases exclude 2005 and 2010 British Election Study data.

A British Election Study (BES) has been conducted at every general election since 1964. Its purpose is to study the long-term trends in British voting behavior; explain election outcomes, party choice and turnout; and to examine the consequences of elections for the operation of democracy more generally [12]. In 2005 the British Election Study team ran an experiment by collecting both face-to-face and the Internet data for the same questionnaire [13]. The CSES questionnaire was included as part of the BES internet survey. In their article Sanders *et al*. present the results of their extensive experiments on the main BES questionnaire. Their analyses showed ‘there are few statistically significant differences between coefficients generated using the in-person and Internet data, and the relative explanatory power of rival models is virtually identical for the two types of data’ [13]. However, since the data were not collected in the approved modes the 2005 BES data are not included in the CSES harmonized dataset. Instead the 2005 British data were collected as part of a self-completion post-electoral supplement to the annual British Social Attitudes (BSA) survey. In 2010 the British Election Study team again collected CSES data using the post-election web panel [14]. As a consequence the third module of the CSES does not include the 2010 BES data in its publically released dataset. Rather than requiring each comparative researcher to combine the BES and CSES datasets individually, wasting time by repeating the same harmonization work and possibly making different coding decisions, we used CharmStats to create a publishable harmonization project that includes syntaxes for SPSS and Stata and can be cited [15].

### How the CSES harmonizes data

The CSES works to implement a common set of questions for participating countries’ post-election studies. Producing high-quality and transparent data harmonizations for statistical analysis and replication requires familiarity with the source and target dataset collection methods and sampling frames. Simply pooling the variables as measured is often not possible due to individual national standards, e.g. education variables reflect national structures that are not equivalent. Consequently, some CSES variables are ‘output harmonized’. Output harmonization is a necessary and routine process; however, it impacts on the cross-national comparability of harmonized measurements as well as the data’s quality and replicability [16]. The process of output harmonization occurs in three major steps: assessing the data at the collection level, assessing the variables at the measurement level, and documenting the process of concept harmonization by recording the exact transformation of the source variable into the harmonized structure (target variable). We review each of these steps below.

The first task is to ensure that all the variables to be used are available in both the target and source datasets. Then the researcher must familiarize himself with the structures of the source and target datasets to be harmonized. This means having information on the underlying populations, the study’s sampling procedures, whether the entire population of interest is covered by the source data and whether the source population of interest can be matched to the target population of interest. Researchers responsible for variable harmonizations must ensure the comparability and compatibility of the data itself, in addition to checking the harmonized variables for any systematical drop-outs that may occur. The third step is harmonizing the measures. The researcher must know the question wording of the source and target variables and response categories as provided to respondents. Guided by the questions and response options a researcher will most likely start with linking identically-worded response categories in both questions. Next would be to collapse individuated response categories in the source variable–in our example ‘divorced’ and ‘separated’- with its multi-category response option counterpart in the target variable ‘divorced or separated’. Afterwards the researcher will focus on response categories that are available in one variable but not in the other, or have slightly different wording in the response options. For example the 2010 BES marital status response category “living with a partner” must be collapsed into the most conceptually equivalent CSES category “married or living together as married”.

In the final step the researcher must document this harmonization process to ensure transparency and replicability for future use; the researcher must write an overview of the source and target studies, their underlying populations, sampling procedures etc., the question wording and response options for each source and (when relevant) target variable, the conceptual mechanism captured by the target variable, as well as the decision criteria for recoding source variable response options. To ensure a high level of transparency, the researcher must detail all the coding decisions, providing the full information necessary to replicate the research.

Finally to illustrate the practicalities in documenting harmonization, in the example above it is important to note that we harmonize data for a project in which missing values (such as refused, skipped, don’t know or missing) are all coded to ‘system missing’ in the appropriate statistical software package. Likewise, some values mentioned in the CSES or the BES codebooks are not included in the example. For instance, the CSES value 5 (labelled as “see election notes”) allows researchers to code marital statuses not covered by the fixed categories 1 to 4, such as “long-term relationship” in the 2008 Austrian subsample. Since the BES does not include an outstanding category that does not fit into the standard CSES categories, the CSES code 5 remains unused and is thus not shown in the mapping (Fig 1).

## Materials and Methods

### Publishing and citing with QuickCharmStats

Using the QCS workflow and features, we propose the following formats as standards for published harmonizations. We call these digital harmonization projects. The QCS digital harmonization project contains metadata on multiple levels. The Project area describes the project itself and its contents. In the Concept area users insert the concept definition(s) and links to relevant literature or related studies and publications. Next, the target and source variable metadata are provided in their own sections. The syntaxes for the harmonization, in SPSS and Stata formats, are included in the report. The last section is for graphs generated by QCS, displaying the harmonization mapping in an easy-to-read visual format. We designed the QCS workflow and report so each section has a level with its own type of metadata. Our example digital harmonization project contains the following information:

Project: Project level data includes the harmonization project name and information on the intent of the project. The first challenge was determining how projects should be named. The information used to construct a project name is important since, in future, finding the right project will depend, in part, on the information included in the name. Naming projects is a challenge because one researcher could be searching for marital status measures, another could be looking for British data, and yet another could be seeking CSES data. In our view, the best way to ensure that future users can find the information is to standardize the naming of harmonization projects. The name of the concept and the target and source datasets used in a project name will be converted into keywords for search engines when deposited for publication.

As a best management practice we recommend individual CharmStats harmonization projects build their names ordered by the following information: (1) the concept to be harmonized, (2) the name of the target variable’s study (or its acronym), and (3) the name of the source variable(s) study/ies or acronym(s). In this example the project name is ‘Marital Status Comparative Study of Electoral Systems Module 3 to British Election Study 2010’. If we were harmonizing two source variables to a single target variable, the project name would be ‘Marital Status Comparative Study of Electoral Systems Module 3 to British Election Study 2010 and British Election Study 2005’. More than three source variables to a single target variable would be named ‘Marital Status Comparative Study of Electoral Systems Module 3 to British Election Study 2010, British Election Study 2005, et al.’ CharmStats’ project notes provide space for short descriptions about the project. Other project level information includes the submission and publication dates for the project and the names of the author(s). Project end use notes provide specific or more technical information on how the variable was used.

Harmonization Syntax: QCS produces syntaxes ready for use either in SPSS or Stata. Including the syntax in the documentation should reduce the time and effort needed to replicate others work or to compare harmonization structures across published results. Future versions of QCS and other CharmStats products will provide the recoding in additional popular proprietary statistical package syntaxes.

Concept: This area records the name of the concept to be harmonized and the definition of the concept (or compatible/competing definitions) that guided variable operationalization. Space is provided to write comments on the concept as used in the research as well as the names of and hyperlinks to relevant literature, publications or studies that informed the recoding. Providing extra space for the concept definition and researcher comments should increase clarity and transparency in replication.

Whether future researchers replicate the concept as operationalized in that particular project or whether they are inspired to create new measures, the first step is to provide detailed documentation. This can be illustrated by examining the conflation of multiple concepts within the ‘marital status’ response options. Although the target and source variables both are named ‘marital status, one very narrow but valid definition of marital status could be ‘the condition of being married or unmarried’ [17] This would limit response categories to ‘single’ or ‘married’. In practice, social surveys collect descriptive information from single people (divorced, widowed or have married) and from married people (living apart but still married). To continue with this necessary pedantry, marital status responses options often include ‘living with a partner’ or ‘living together as married’ options that best represent the concept of cohabitation: ‘a living arrangement in which an unmarried couple lives together in a long-term relationship that resembles a marriage’ [18]. While this is common in practice, it is still conflating two different concepts into one measure and we note this in our concept comments. Further, we note the available response categories cannot capture same-sex civil partnerships or changes in marriage laws as they relate to same-sex couples. Space for precise definitions and the rationale for coding decisions are important for theoretical reasons and secondary reuse. For instance, sociological investigations into marriage and cohabitation might see greater differences between the options “being married”, “living with a partner” and “living together as married” than a political scientist. For researchers using European data, in the Netherlands or France the status of ‘legal partnership’ implies a different legal status than simply ‘living together with a partner’ or than being ‘married’ [19, 20], while cross-national political scientists might aim for an overall comparability of the marital status control variable between different election studies.

Target and source variables: Target and source variable sections in the report contain similar metadata and information. These include the target and source variable names and labels, the sampling and measurement levels, the question wording and response wording options and values. The source variable also lists the name of the ‘instance’ in which it is used within the QCS software. When more than one source variable is harmonized to a target variable, each gets a unique ‘instance’ name to differentiate it from other source variables within the same project. Space is provided so researchers can include the name of study dataset and links to the study’s (or studies’) persistent identifier(s). In our example we provide the DOI for the CSES study, however at the time of writing, the 2010 British Election Study had not yet deposited their data. Instead, the BES data is available on the study’s website. We list the BES data url and date last accessed in lieu of a DOI. This is an attempt to capture the versioning of studies not submitted for preservation with a data archive. Finally, both target and source metadata provide space where researchers can include relevant comments. CharmStats users may import existing variables or they can create their own target variables and document that process within the project; the comments section is where researchers can elaborate on other details or construction of the measure. The researcher needs to document, and thus explain, why she collapsed the BES “living with a partner” category into the CSES category “married or living together as married” as opposed to keeping the BES category separate (e.g. using the CSES category 5 that can be modified to be any response option in the CSES). Depending on the research question, unmarried couples might make a difference in the outcome of the analysis and thus impact the research findings. Providing a rational for the collapse of certain categories can be necessary information to achieve full transparency.

Future versions of QCS and CharmStats products will allow users to import certain forms of DDI so that, in addition to the study metadata, users may import question and survey metadata directly into their projects. In addition to including the question wording, we recommend researchers include additional question-level metadata when it is relevant to how the variable should be used in replication, for instance, noting routed questions, interviewer instructions or explaining the skip pattern structure.

Graph: QCS includes a graph generator. Fig 1 shows the marital status harmonization mapping. Users may inspect these graphs, insert them into presentations and include them in their published harmonization. The graphs allow users to spot coding errors before running the syntax, to clarify the coding of collapsed response options; and to make it easier to compare between different harmonizations structures across (and/or within) datasets. Researchers can evaluate data loss between alternative harmonizations by visually comparing the number and types of collapsed categories.

### Peer review and publishing

In her article, Replication as Regulation, Fowler wrote that replication provides the basis for a cumulative body of work using similar methods and data but that ‘the amount of information sharing is likely to be suboptimal without some sort of incentive or requirement to bring it about’ [21]. CharmStats software products are designed to provide a digital publication pathway for harmonization work.

Until the development of CharmStats products, collecting the necessary metadata to fully and transparently document variable harmonization work was simply too time consuming and, although it would be virtuous work, there was no career pay-off for the hours spent assembling the information needed for precise replication. At present the intellectual contributions of data harmonizers go unrecognized and their work is not easily citable. Even if a researcher managed to document their variable harmonizations there is no place for them to publish it. QuickCharmStats organizes a researcher’s work in a format pre-prepared for the peer-review process. If a harmonization documentation project is accepted for publication it will receive a permanent identifier that can be listed as a reference when citing researchers’ original harmonization work in a publication. Clicking the permanent identifier link will take the reader directly to the published harmonization documentation–including the recoding syntax–making it freely available, easily findable as well as replicable and citable by other researchers.

Researchers who wish to submit a digital harmonization project for publication can download the peer-review submission report template from the GESIS CharmStats website. The completed report and the graph should be emailed to: charmstats@gesis.org. If the submission does not meet the standards of peer review the researcher will be given precise instructions on what to include in order for it to be accepted. Once accepted the submitted digital harmonization will be assigned a DOI and published on a GESIS website or deposited into an online harmonization library, free of cost.

Finally, we propose a referencing guideline for CharmStats harmonization citations. As discussed above in the project naming rationale, standardizing the project titles will facilitate finding and accessing harmonizations and will thus promote secondary use. We adapted current citation guidelines for datasets to fit digital harmonization projects, including the project’s hyperlink, date of publication, version or DOI and the date the author last accessed the documentation. We provide an example based upon the marital status harmonization.

Winters K, Netscher S Marital status Comparative Study of Electoral Systems Module 3 to British Election Study 2010. [harmonization] Cologne, Germany: GESIS [distributor] 2015. (Accessed 4 January 2016). http://dx.doi.org/10.7802/1160.

## Conclusions

QuickCharmStats is the digital tool that can fulfill the scientific community’s variable harmonization documentation and replication needs. QCS was designed to reduce the time, costs and effort associated with documenting harmonizations and to increase the secondary use of data. It is a proven advantage over the existing hand-typed alternatives. Through the use of metadata importation, QCS allows researchers to quickly assemble the information required for others to replicate their harmonized variables and—following peer review—provides a means for publishing and citing their work within journal articles. It is our hope that this new digital tool will make a substantial contribution to increased data reuse. We started out investigating how best to meet the standards of scientific replication in data harmonization by determining the necessary and sufficient information required to replicate research data in terms of matching decision in the context of variable categories, and thus increase the transparency of research findings. Moreover, we aimed to provide a means to credit to researchers whose intellectual contributions in creating new and/or harmonized variable have been heretofore unacknowledged.

Other CharmStats products are in development. The next release, CharmStats Pro (CS Pro), will operate using a dedicated database server designed for use by teams of researchers working on large scale studies or surveys, such as the European Values Survey (EVS) and The International Social Survey Programme (ISSP). CS Pro is scheduled for release in 2016 and will allow for importing and exporting metadata using various versions of DDI. In addition, a web-based harmonization library, compatible with CharmStats products and the full range of DDI formats, is in development. GESIS was awarded EU funds to create an Online Library of Harmonizations, a website where researchers can deposit CharmStats harmonization projects and study codebooks (first for peer review and then publication) so that others can search for and download their digital harmonization projects for replication. GESIS–Leibniz-Institute for the Social Sciences is Germany’s largest infrastructure institute for the social sciences. It provides research-based services and consulting on all levels of the scientific process. GESIS is committed to creating free and open-source digital infrastructure for long-term use and scientific impact. Therefore, the maintenance of CharmStats products and development of further online products, product up-dates and support, and product development are secure.

In CS Pro researchers will be able to include technical notes, add literature citations that informed their coding, space to record their decision guidelines and the rationales behind them, to note anomalies in the data and the strategies they used to deal with them, as well as the ability to attach hyperlinks to other codebooks, questionnaire documents, or relevant study notes. Large scale studies will be able to generate and upload digital codebook by collating individual digital harmonization projects. Once deposited in the online harmonization library they will be searchable, easily accessible and freely available, and will provide researchers worldwide with the syntax they need for precise replication. Our long-term goal is for principal investigators and administrators on all large-scale studies to convert their documentation practices away from.pdf or.txt codebooks and into digital codebooks that can be deposited in and retrieved from the Online Harmonization Library at GESIS (scheduled to go online in 2018).
